# Mapping the landscape of treatable inborn errors of metabolism: a systematic gene-level evaluation based on the ICIMD classification

**DOI:** 10.1186/s13023-026-04486-0

**Published:** 2026-07-03

**Authors:** Tabeer Fatima, Jalal Khan, Fatma Al-Jasmi, Hiba Alblooshi

**Affiliations:** 1 Department of Genetics and Genomics, College of Medicine and Health Sciences, United Arab Emirates University, Al Ain, United Arab Emirates; 2 Division of Genetic Metabolic, Department of Pediatrics, Tawam Hospital, Al Ain, United Arab Emirates

**Keywords:** Inborn errors of metabolism, Inherited Metabolic Disorders, ICIMD, Targeted therapy, Treatable IEMs, Rare diseases, Metabolic Treatabolome, Treatable ID, DDIEM, IEMbase.

## Abstract

**Background:**

Inborn errors of metabolism (IEMs) are among the most clinically actionable groups of rare genetic diseases, yet therapeutic knowledge remains distributed across multiple databases, complicating consistent identification of treatable IEMs.

**Methods:**

In this study, we performed a cross-database analysis to characterize the current therapeutic landscape of IEM genes using GeneReviews as a clinical reference. Gene symbols from GeneReviews, Metabolic Treatabolome, Treatable ID, and the Drug Database for Inborn Errors of Metabolism (DDIEM) were harmonized to HGNC nomenclature and mapped to the International Classification of Inherited Metabolic Disorders (ICIMD). IEM genes were categorized as associated with targeted or supportive treatment based on a GeneReviews-derived benchmark.

**Results:**

Among 656 ICIMD-defined IEM genes in GeneReviews, 155 (23.6%) were associated with targeted therapies and 501 (76.4%) with supportive treatment. Cross-database comparison demonstrated limited concordance, with only 36 genes associated with targeted therapy shared across Metabolic Treatabolome, Treatable ID, and DDIEM, reflecting heterogeneous representation of treatable IEMs across databases. Targeted therapies were more common in intermediary metabolic pathways, including fatty acid, tetrapyrrole, and vitamin/cofactor metabolism, whereas mitochondrial and complex cellular disorders were predominantly associated with supportive management. Moreover, 53% of the ICIMD-defined IEM genes remain unrepresented in structured databases.

**Conclusions:**

Our study defines a set of IEM genes for which targeted therapies are available and demonstrates that treatment information is largely fragmented across databases. This set of IEM genes may facilitate the interpretation of genomic findings and aid the prioritization of IEMs for genomic screening and clinical decision-making.

**Supplementary Information:**

The online version contains supplementary material available at https://doi.org/10.1186/s13023-026-04486-0.

## Introduction

Rare genetic diseases collectively impact millions globally, but only 5% have available treatment [1–4]. Among these diseases, Inborn Errors of Metabolism (IEMs) are one of the most clinically actionable groups, as treatment may directly modulate the underlying biochemical pathway rather than merely alleviating symptoms. Consequently, early diagnosis is crucial, as it has immediate implications for clinical management and is essential for improving clinical outcomes. For many IEMs, delayed treatment may result in preventable morbidity/mortality or irreversible complications [5–12].

Therefore, to consistently define IEMs, the International Classification of Inherited Metabolic Disorders (ICIMD) provides a mechanism-oriented classification that delineates disease within the IEM spectrum [13]. In parallel, distinguishing the nature of therapeutic interventions is essential for understanding the overall treatment landscape of these diseases. Within IEMs, therapeutic interventions differ substantially in clinical relevance. Some treatments target the underlying biochemical defect and may alter the disease course, either by reducing toxic substrate accumulation, replacing deficient products, or replacing the missing enzyme. Strategies include dietary restriction, prevention of catabolism, alternative pathway therapy, dialysis, enzyme replacement, cofactor supplementation, substrate reduction therapy, chaperone therapy, and in selected cases, stem cell transplantation, liver transplantation or gene therapy, whereas others symptomatically address downstream manifestations [14]. 

To formalize this distinction, a recent GeneReviews-based study defined targeted therapy as an intervention that acts on the underlying molecular mechanism of a disease and has the potential to modify disease course, including curative outcomes in some instances. Such therapies may be recognized only after genetic diagnosis. This definition enables systematic differentiation between mechanism-based therapies and symptomatic management [15]. 

Despite the clinical importance of this distinction, treatment-related information remains distributed across multiple databases, making it difficult to consistently determine which IEMs have mechanism-based therapeutic options. As a result, the presence or absence of a targeted treatment for a given IEM may depend on the database consulted. This complicates the interpretation of genetic findings and the identification of treatable IEMs. Several structured databases catalog therapeutic information for IEMs, including Treatable ID, Metabolic Treatabolome, and the Drug Database for Inborn Errors of Metabolism (DDIEM). These databases compile treatment data using different scopes, curation strategies, and data structures. In addition to structural heterogeneity, the level of therapeutic evidence differs across databases, ranging from experimental or literature-reported interventions to clinically established treatments. A consistent reference grounded on established therapeutic practices is therefore required to interpret treatment information across databases. Furthermore, different databases capture overlapping but distinct subsets of IEMs. Integrating information across databases is therefore necessary to obtain a comprehensive view of treatment availability [14, 16–18]. 

To address the need for a consistent clinical reference, an ICIMD-based IEM classification dataset was generated to identify the representation of IEM genes across all databases. The curated IEM genes were subsequently categorized by treatment type, including targeted therapies and symptomatic management, using GeneReviews. GeneReviews was selected as the anchor resource because its management recommendations primarily reflect established therapeutic practice. 15,19.

We therefore mapped IEM treatment landscape across four databases, GeneReviews, Metabolic Treatabolome, Treatable ID, and DDIEM, using GeneReviews as the reference. Our work enabled systematic evaluation of treatment coverage and identification of gaps across databases.

## Methods

**Fig. 1:**
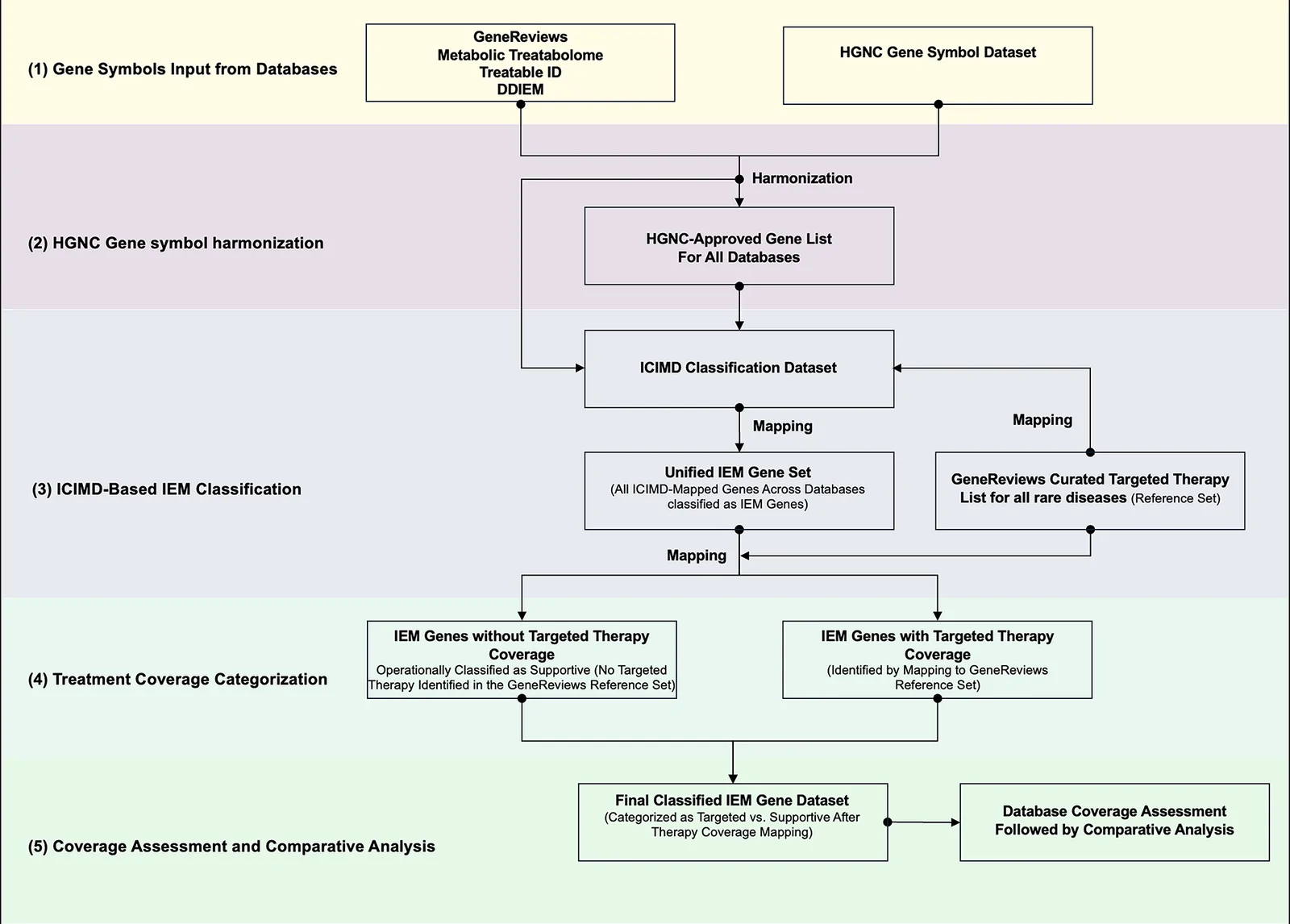
Schematic workflow for HGNC harmonization, ICIMD-based IEM genes classification, and treatment categorization

An overview of the study workflow, including data integration, gene symbol harmonization, ICIMD-based IEM genes classification, and treatment categorization, is provided in Fig. 1. The complete HGNC-harmonized gene lists and unified IEM gene sets are provided in Supplementary Tables 1–3.

1. **Gene Symbol Input from Databases**.

Gene symbols were extracted from publicly available datasets and primary publications of Metabolic Treatabolome, Treatable ID, DDIEM, and GeneReviews. In addition, a previously published GeneReviews-curated targeted therapy list was obtained and used as a benchmark reference set for categorizing IEM genes by treatment [14, 15, 17–19]. All analyses were conducted at the gene-symbol level.

2. **HGNC Gene Harmonization**.

All extracted gene symbols were harmonized to the current HUGO Gene Nomenclature Committee (HGNC) approved nomenclature (last accessed 24 September 2025). Historical symbols and aliases were mapped to their corresponding approved HGNC symbols [20, 21]. Duplicate entries arising from cross-database overlap or symbol updates were removed. Following harmonization, the HGNC-approved gene list was generated, representing all genes from all databases. These harmonized lists were used for all downstream mapping processes.

3. **ICIMD-Based IEM Classification**.

To restrict the analysis to Inborn Errors of Metabolism (IEMs), harmonized gene symbols from all databases were mapped to the ICIMD classification dataset [13]. Genes successfully mapped to ICIMD were defined as IEM genes. Genes not corresponding to ICIMD-defined IEMs were excluded from further analysis. The same filtering procedure was applied to the GeneReviews curated targeted therapy reference set to exclude entries other than IEMs (Supplementary Table 4).

4. **IEM Gene Set Consolidation**.

A unified IEM gene set was generated by taking the union of all ICIMD-mapped genes across all databases. This unified IEM gene set served as the basis for the IEM gene landscape for treatment coverage analysis. Treatment coverage was identified by performing a set intersection between the unified IEM gene set and the GeneReviews curated targeted therapy reference set for IEM genes. Genes present in both sets were considered to have targeted therapy.

5. **Treatment Coverage Categorization**.

Based on the GeneReviews curated targeted therapy list, IEM genes from all databases were categorized into two groups:

1. **IEM genes with targeted therapy coverage**, defined as genes present in the GeneReviews curated targeted therapy reference set.
2. **IEM genes without targeted therapy coverage**, operationally classified as supportive treatment genes, defined as ICIMD-mapped IEM genes not identified in the targeted therapy reference set.
3. **Coverage Assessment and Comparative Analysis**.

Comparative analyses were performed across all databases, and identified IEM genes based on ICIMD, followed by treatment categorization using a targeted therapy reference set from GeneReviews. A unified IEM gene set was mapped across all 24 ICIMD disease categories. The final classified IEM gene dataset was used to evaluate database-level coverage of targeted therapy versus supportive treatment information.

7. **Statistical Analysis and Visualization**.

All data processing, set operations, and visualizations were performed in Python using the pandas and matplotlib libraries.

## Results

### ICIMD-based filtering of IEM genes across all databases

**Fig. 2:**
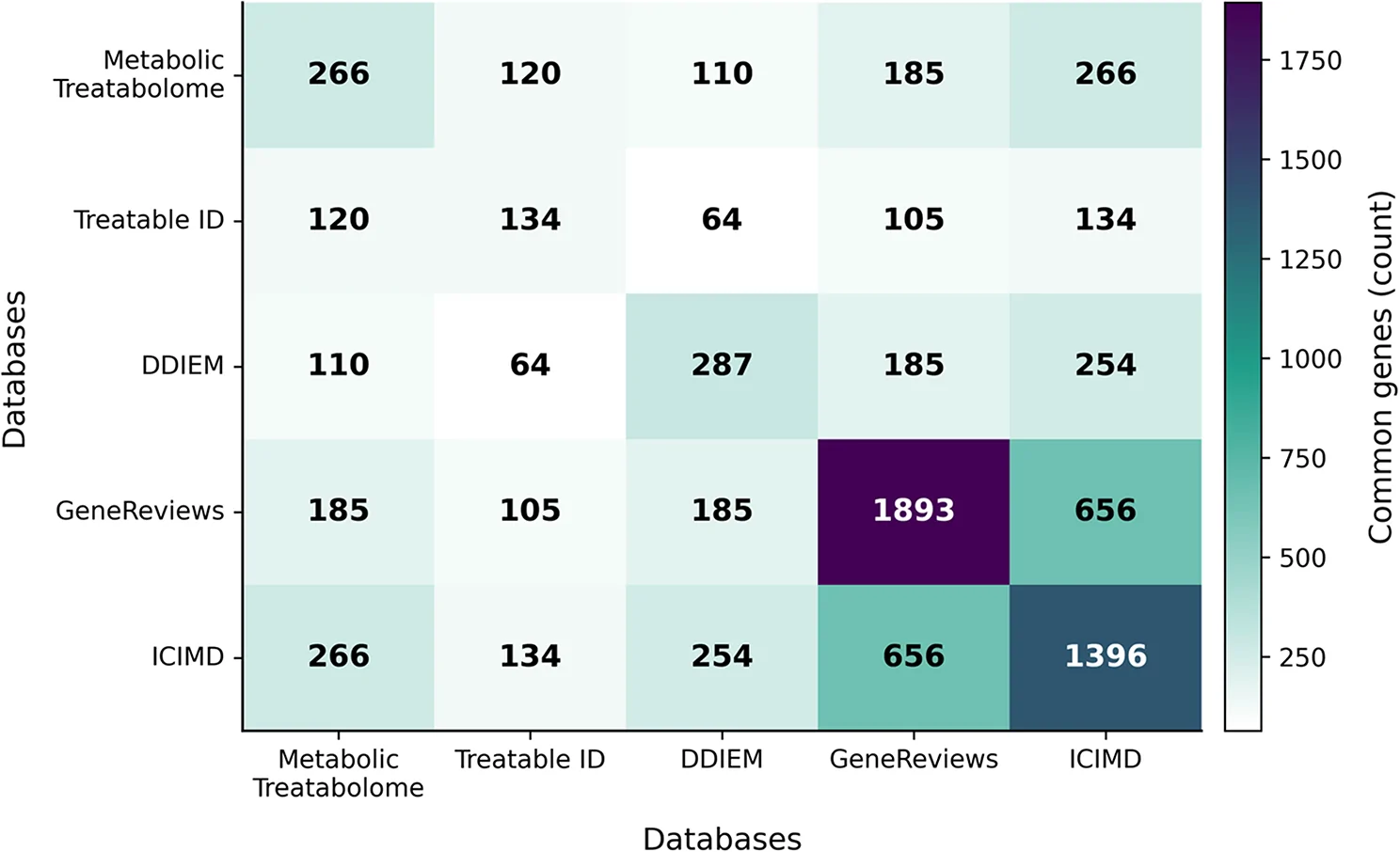
Pairwise overlap of genes across rare disease databases. Heatmap showing the number of shared genes between Metabolic Treatabolome, Treatable ID, DDIEM, GeneReviews, and ICIMD. Diagonal cells indicate the total number of genes curated within each database, while off-diagonal cells represent pairwise gene overlap. Color intensity reflects the magnitude of shared gene counts, as indicated by the color scale. Gene symbols were harmonized prior to comparison to ensure consistency across databases

Figure 2 summarizes the gene overlap among Metabolic Treatabolome, Treatable ID, DDIEM, GeneReviews, and ICIMD following gene symbol harmonization. The number of curated genes varied substantially across databases, ranging from 134 genes in Treatable ID and 266 in Metabolic Treatabolome to 287 in DDIEM and 1,893 in GeneReviews, while ICIMD contained 1,396 genes (Detailed gene lists are provided in Supplementary Table 1).

Pairwise comparisons revealed varying degrees of agreement among databases. Metabolic Treatabolome shared 120 genes with Treatable ID and 110 genes with DDIEM and shared a larger set of genes with GeneReviews (185 genes). Notably, all 266 Metabolic Treatabolome genes were represented within ICIMD, indicating that this database is entirely confined to ICIMD-defined IEMs.

Treatable ID exhibited a smaller but consistent overlap pattern, sharing 64 genes with DDIEM and 105 genes with GeneReviews, while its entire gene set (134 genes) was also contained within ICIMD. Similarly, a substantial proportion of DDIEM genes aligned with ICIMD (254 of 287 genes), whereas only 185 overlapped with GeneReviews. Furthermore, GeneReviews showed the broadest coverage with 656 genes overlapping ICIMD. IEMs accounted for a major proportion of the GeneReviews rare-disease genes, representing 34.7% of the total of 1,893 genes. Collectively, these results demonstrate heterogeneous coverage of ICIMD-defined IEM genes across databases.

**Fig. 3:**
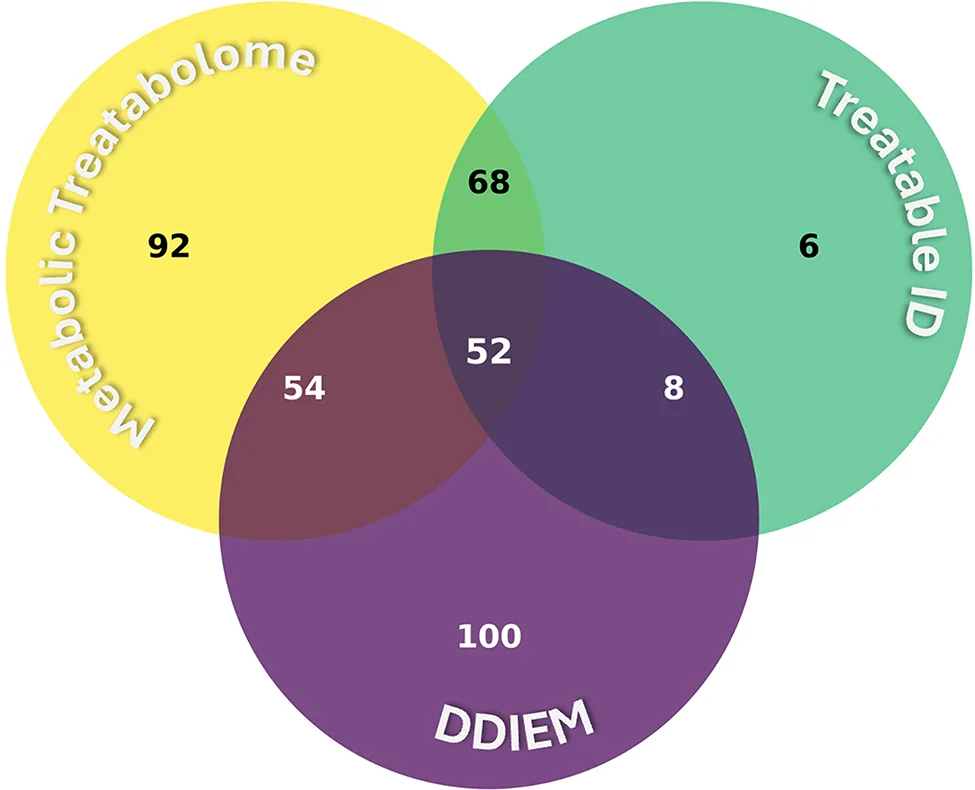
Overlap of ICIMD-defined IEM genes across Metabolic Treatabolome, Treatable ID, and DDIEM after HGNC harmonization and ICIMD filtering, showing limited concordance among IEM-focused databases

To further assess heterogeneity among IEM-focused databases, concordance of ICIMD-defined IEM genes across the Metabolic Treatabolome, Treatable ID, and DDIEM was examined (Fig. 3). Despite their shared objective of cataloging treatable metabolic disorders, the extent of shared gene representation varied across databases. Only 52 genes were common to all three databases, while many genes were unique to individual databases or shared only between two databases. This shows that the databases not only partially overlap but also include different sets of IEM genes. To facilitate systematic comparison despite this discordance, a standardized clinical reference framework based on curated management recommendations was required; therefore, the GeneReviews database was used as the primary source, as it consists of the largest set of IEM genes among all databases (Fig. 2).

### GeneReviews-based categorization of IEM genes across IEM-focused databases

**Fig. 4:**
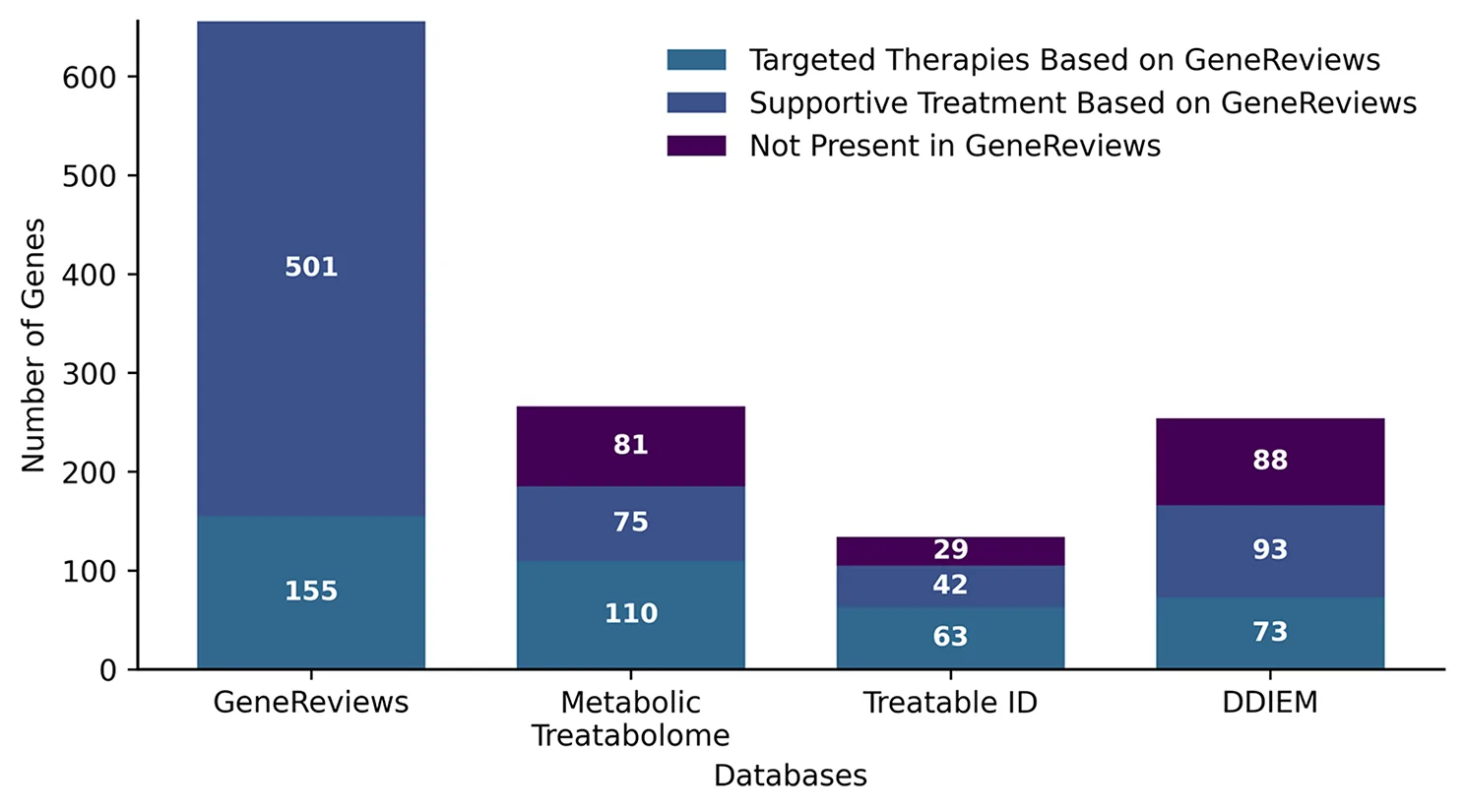
GeneReviews-based treatment categorization across IEM-focused databases. Stacked bar chart showing the number of ICIMD-defined IEM genes categorized according to GeneReviews as having targeted treatment, supportive treatment, or not present in GeneReviews across Metabolic Treatabolome, Treatable ID, DDIEM, and GeneReviews (Detailed gene list is provided in Supplementary Tables 2 and 3). The x-axis represents the databases and the y-axis represents gene counts. Numbers within bars indicate the number of genes in each category

Among the 656 ICIMD-defined IEM genes identified in GeneReviews, 155 were associated with targeted treatment based on our reference set and the remaining 501 with supportive treatment, indicating that supportive management accounted for the majority of documented interventions within GeneReviews (Fig. 4, Supplementary Table 3).

Using this GeneReviews-based categorization, genes present in other databases were evaluated according to whether they corresponded to targeted treatment, supportive treatment, or were absent from GeneReviews. Metabolic Treatabolome contained 110 genes aligned with targeted treatment and 75 aligned with supportive treatment, while 81 genes in this database were not represented in GeneReviews. Treatable ID showed a similar but smaller distribution, with 63 targeted and 42 supportive genes, and 29 genes absent from GeneReviews. In contrast, DDIEM included 73 targeted-treatment genes and 93 supportive-treatment genes, alongside 88 genes not captured in GeneReviews.

Overall, the three IEM-focused databases differed in how their gene content aligned with the GeneReviews as the primary source: Metabolic Treatabolome showed the highest number of IEM genes with targeted therapy, Treatable ID represented a more restricted subset, and DDIEM contained a comparatively larger proportion of IEM genes with supportive treatment as well as the greatest number of genes not covered by GeneReviews.

### Cross-database interpretation of targeted and supportive treatments

**Fig. 5:**
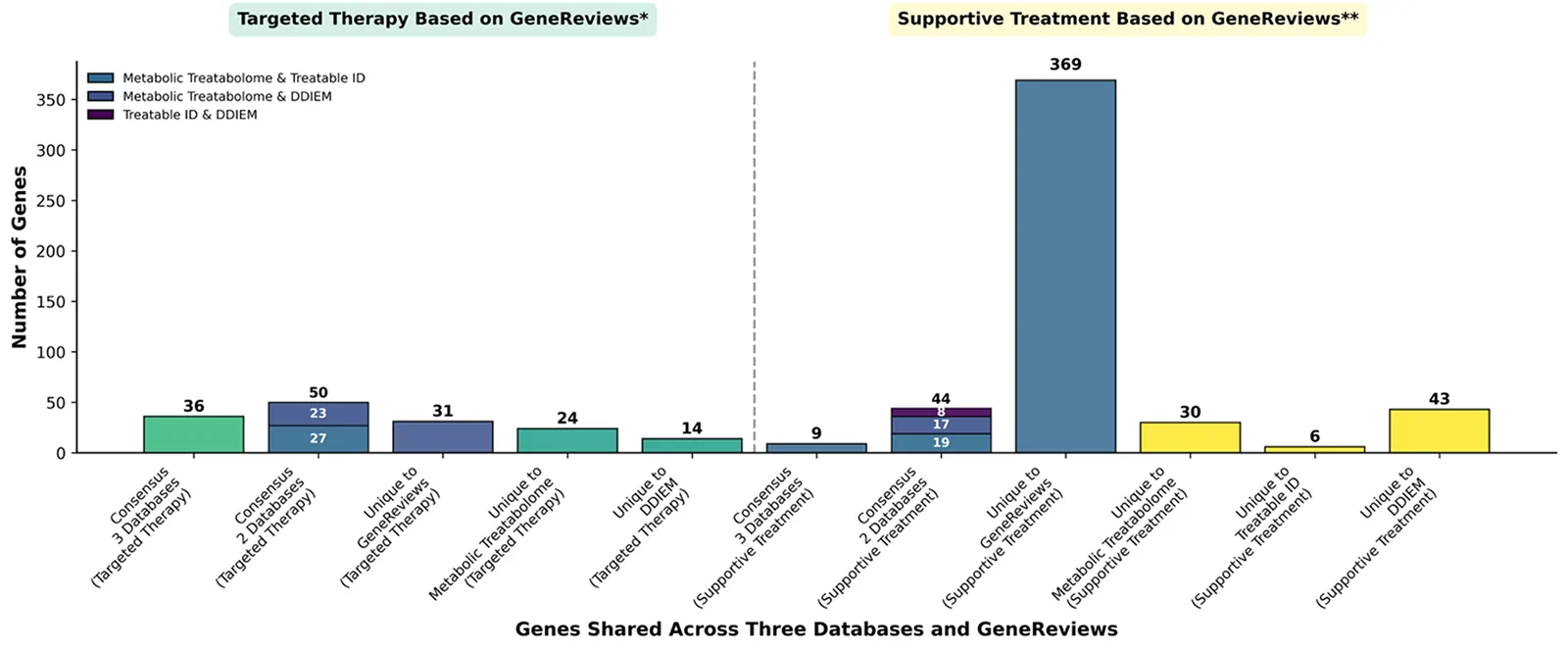
Cross-database distribution of IEM genes with targeted and supportive treatments. Bar plots show the number of IEM genes categorized according to GeneReviews as having targeted treatment (left panel) or supportive treatment (right panel). The x-axis represents gene groups shared across three databases, shared between pairs of databases, or unique to individual databases (Metabolic Treatabolome, Treatable ID, DDIEM, and GeneReviews), and the y-axis represents gene counts. Numbers above bars indicate the number of genes. *Targeted therapy assigned based on the published GeneReviews-derived list; **Supportive treatment assigned to remaining GeneReviews IEM genes

Following the GeneReviews-based categorization, we examined how IEM genes with targeted or supportive treatments were represented across the Metabolic Treatabolome, Treatable ID, and DDIEM (Fig. 5).

For targeted therapy, 36 genes were shared across all three databases. A further 50 genes were shared between two databases, including 27 shared between Metabolic Treatabolome and Treatable ID and 23 shared between Metabolic Treatabolome and DDIEM. Thirty-one genes were unique to GeneReviews, while 24 and 14 genes were unique to Metabolic Treatabolome and DDIEM, respectively.

For supportive treatment, 9 genes were shared across all three databases. Forty-four genes were shared between two databases, including 19 shared between Metabolic Treatabolome and Treatable ID, 17 shared between Metabolic Treatabolome and DDIEM, and 8 shared between Treatable ID and DDIEM. GeneReviews additionally contained 369 supportive-treatment genes not captured in the other databases. Furthermore, certain genes were shared exclusively between GeneReviews and individual databases: 30 with Metabolic Treatabolome, 6 with Treatable ID, and 43 with DDIEM.

### Treatment landscape across ICIMD categories

**Fig. 6:**
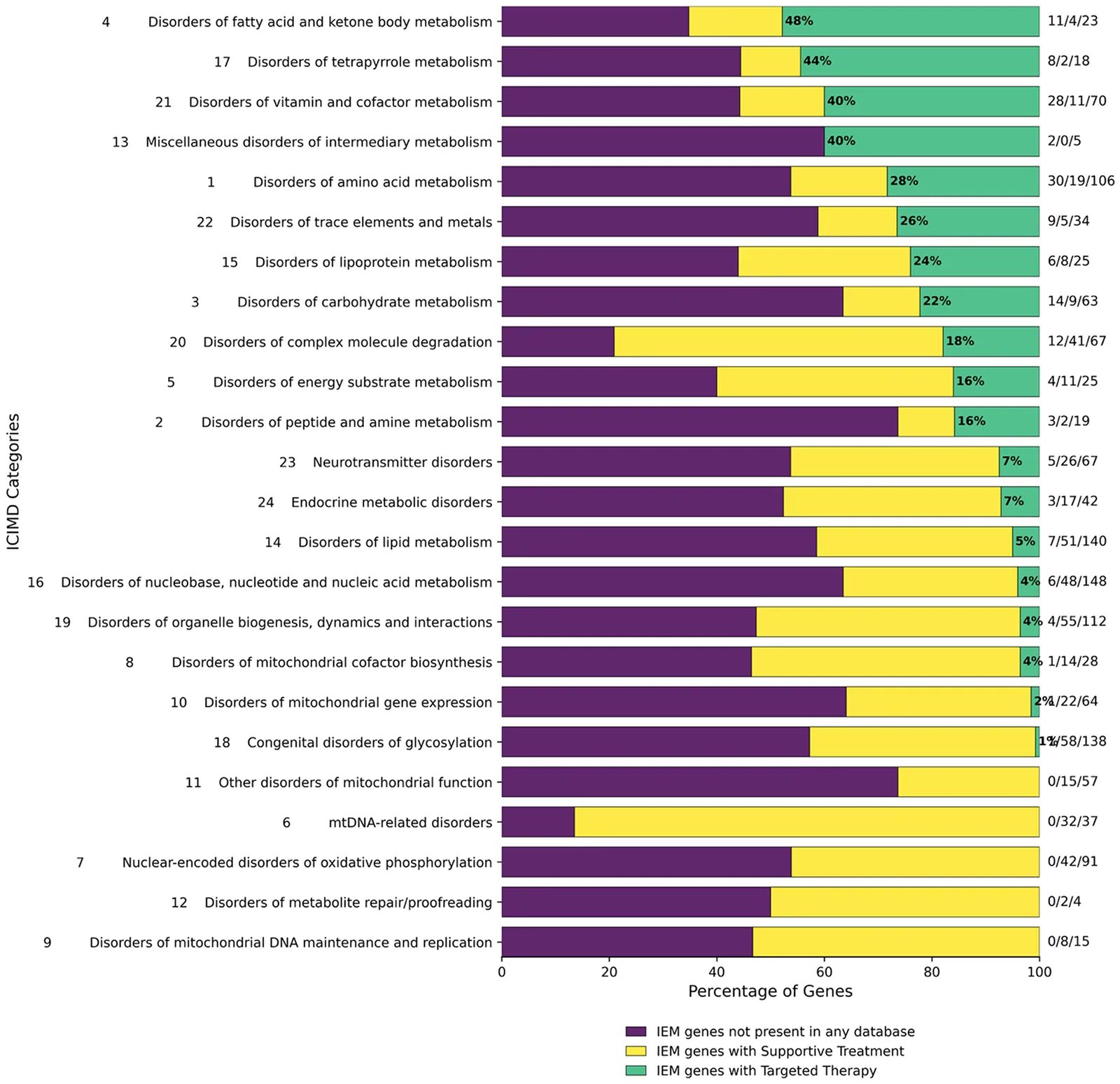
Treatment distribution for IEM genes across ICIMD categories. Stacked horizontal bars show the proportions of genes in each ICIMD category that are distributed as IEM genes with targeted treatment, supportive treatment, or with no treatment information across the surveyed databases. The x-axis represents the percentage of genes, and the y-axis lists 24 ICIMD categories. Percent labels indicate the fraction of genes with targeted treatment within each category, and the numbers on the right indicate counts of targeted/supportive/absent genes, respectively

The distribution of IEM genes with targeted therapy, supportive treatment, and those without documented management was evaluated across 24 ICIMD categories (Fig. 6).

Genes associated with targeted therapy were most frequent in disorders of fatty acid and ketone body metabolism (48%), tetrapyrrole metabolism (44%), vitamin and cofactor metabolism (40%), and miscellaneous intermediary metabolism (40%). Intermediate proportions (20–30%) were observed in amino acid metabolism (28%), trace element and metal disorders (26%), lipoprotein metabolism (24%), and carbohydrate metabolism (22%). Lower proportions (10–20%) were identified in complex molecule degradation (18%), energy substrate metabolism (16%), and peptide and amine metabolism (16%). Very low representation (< 10%) occurred in neurotransmitter disorders (7%), endocrine metabolic disorders (7%), lipid metabolism (5%), nucleobase, nucleotide and nucleic acid metabolism (4%), organelle biogenesis and dynamics (4%), and mitochondrial cofactor biosynthesis (4%). Minimal representation was observed in mitochondrial gene expression (2%) and congenital disorders of glycosylation (1%), while no genes with targeted therapy were identified in mtDNA-related disorders, nuclear-encoded oxidative phosphorylation disorders, metabolite repair/proofreading disorders, or mitochondrial DNA maintenance and replication.

IEM genes with supportive treatment were distributed across most ICIMD categories and commonly represented the predominant management approach when targeted therapies were limited. This pattern was particularly evident in disorders involving complex cellular and mitochondrial processes, where interventions were largely supportive rather than mechanism-directed. In several mitochondrial-related categories, including oxidative phosphorylation, metabolite repair/proofreading, and mitochondrial DNA maintenance, IEM genes were almost exclusively associated with supportive treatment when present.

## Discussions

Our study evaluated treatment representation for inborn errors of metabolism (IEMs) across major IEM-focused databases and GeneReviews to identify substantial gene-level discordance among them. To enable reliable cross-database comparison, we first harmonized gene symbols to the current HGNC-approved nomenclature. Without this harmonization, comparisons across all databases could retrieve partial, duplicated, or misaligned gene entries due to previous symbols and aliases, which are known to hinder consistent data mining across biomedical resources [22]. 

Using the ICIMD classification as a mechanism-based framework, 656 of the 1,893 curated GeneReviews genes were identified as IEM genes, indicating that approximately one-third of GeneReviews entries correspond to metabolic disorders. Given that Metabolic Treatabolome and Treatable ID have already adopted the ICIMD classification, our mapping of DDIEM to ICIMD showed that 254 of 287 genes aligned with ICIMD-defined IEMs, demonstrating substantial overlap with the metabolic disease spectrum.

After we have established a unified IEM gene set, treatment categorization analysis revealed that only 155 of 656 IEM genes in GeneReviews were associated with targeted therapies, including dietary interventions, cofactor supplementation, enzyme replacement therapy, substrate reduction therapy, chaperone therapy, transplantation-based approaches, and gene-based therapies (Supplementary Table 5) whereas 155 IEM genes in GeneReviews were associated with targeted therapies, whereas the majority (501 genes) were linked to supportive management. This imbalance indicates that most metabolic diseases are still managed through symptomatic or downstream interventions rather than by correcting the primary molecular defect [23]. 

Cross-database comparison demonstrated heterogeneous representation of IEM genes. Metabolic Treatabolome contained a higher proportion of IEM genes for which targeted therapies are available; Treatable ID represented a selective subset focused on IEMs with intellectual disability; and DDIEM included a broader range of genes for which supportive treatments are available. Only 36 genes with targeted therapies were shared across all databases, whereas many were unique to individual databases. Notably, 31 genes with targeted therapies were identified exclusively in GeneReviews and were absent from the IEM-focused databases, indicating that clinically actionable information may reside outside specialized metabolic knowledgebases. Conversely, 147 genes were present in IEM-focused databases but not captured in GeneReviews, many of which reflect conditions managed primarily through symptomatic or literature-reported interventions; detailed clinical evaluation of these cases was beyond the scope of the present analysis. Together, these observations emphasize fragmentation and incomplete coverage of therapeutic knowledge across IEM-focused databases [18]. 

To understand whether treatability reflects biological properties of metabolic pathways, treatment distribution was examined across ICIMD categories. Categories with the highest proportions of targeted therapies, fatty acid and ketone body metabolism, tetrapyrrole metabolism, and vitamin/cofactor metabolism correspond to disorders in which the biochemical defect can be directly modified by substrate restriction, metabolite supplementation, or enzyme replacement. This distribution indicates that diseases involving defined metabolic blocks are particularly amenable to mechanism-directed treatment strategies [6, 24]. 

The scarcity of targeted therapies in mitochondrial and organelle-related categories suggests reduced pharmacologic tractability of disorders involving multi-component cellular systems. Rather than the accumulation of a single measurable metabolite, these conditions frequently involve impaired energy production or structural cellular dysfunction, and consequently, current treatment approaches remain predominantly supportive rather than disease-modifying [25–27]. 

Overall, this pattern indicates that therapeutic tractability is closely linked to pathway architecture: disorders of intermediary metabolism are more likely to be directly treatable, whereas diseases affecting complex cellular systems remain less amenable to targeted therapies, with emerging treatments currently limited to a small subset of conditions within this group.

The treatment categorization used in this study was derived from the GeneReviews-based definition of targeted therapy and was applied as a comparative framework rather than a clinical classification. Individual interventions may reasonably be interpreted differently by experts, particularly when partial biochemical improvement does not translate into uniform clinical benefit. Moreover, in many IEMs, therapeutic response varies according to phenotype severity and clinical presentation: patients with severe disease may show limited response, whereas those diagnosed earlier or with milder phenotypes may benefit from the same intervention. This consideration applies to both targeted and supportive management; therefore, gene-level categorization reflects the availability of a therapeutic strategy rather than expected effectiveness in every patient.

Supportive treatment was documented across nearly all ICIMD categories, yet a substantial proportion of IEM genes lacked documented management in IEM-focused databases; specifically, 53% of ICIMD defined IEM genes were not represented in any database, including GeneReviews. This indicates that symptomatic care is frequently applied in clinical practice but is not consistently captured in structured databases, reflecting both limited disease-modifying options and incomplete representation of routine clinical management [18]. 

Evaluating treatment availability at the gene level aligns with emerging diagnostic and newborn-screening frameworks based on genomic testing. A population study from the Qatar Genome Program reported that approximately 1 in 13 individuals carries a pathogenic variant in a treatable IEM gene set derived from Treatable ID [28]. By providing a set of 155 IEM genes with targeted therapies, the present analysis expands the reference framework and may support carrier-burden estimation and prioritization of disorders for genomic screening strategies.

Importantly, the study also serves as a practical guide: when information on targeted therapy is absent, supportive management strategies for a given IEM can often be identified across multiple IEM-focused databases. The observed discordance across databases highlights the practical necessity of integrating treatment knowledge to improve clinical actionability.

This study has limitations. The analysis was gene-based and did not consider variant-specific or phenotype-dependent treatment responses. The strength of evidence across databases was also not evaluated. Furthermore, the study was limited to treatment information available in the selected databases and may not fully capture recently reported therapeutic approaches or newly recognized treatable IEMs described in the broader literature. Future work should incorporate graded levels of evidence and phenotype-specific treatment annotations and updated literature-based curation to better reflect clinical decision-making.

The observed fragmentation of therapeutic knowledge further highlights the need for harmonized annotation frameworks and scalable curation strategies. Automated approaches, including artificial-intelligence assisted text mining combined with expert review, may enable continuous updating of therapeutic knowledge and reduce discrepancies between clinical practice and structured databases [14]. By defining a unified set of IEM genes with targeted therapy, the present analysis provides a framework that may directly inform prioritization of disorders in genomic screening programs.

## Conclusion

This study presents an ICIMD-guided, gene-level comparison of treatment representation across major databases for inborn errors of metabolism. Treatment information was inconsistently represented across databases. Although a subset of IEM genes was associated with targeted therapies, supportive treatment remained the predominant documented approach, and a substantial proportion of ICIMD-defined IEM genes were not represented in existing databases.

The identified set of IEM genes with targeted therapies may assist genomic interpretation and prioritization of treatable disorders in diagnostic and screening contexts. Continued harmonization and systematic curation of therapeutic information will be important to improve the consistency and clinical utility of IEM-focused databases.

## Supplementary Information

Below is the link to the electronic supplementary material.

Supplementary Material 1

## Data Availability

The data that support the findings of this study are available in the supplementary material of this article.
